# Genetic information improves the prediction of major adverse cardiovascular events in the GENEMACOR population

**DOI:** 10.1590/1678-4685-GMB-2020-0448

**Published:** 2021-06-11

**Authors:** Maria Isabel Mendonça, Eva Henriques, Sofia Borges, Ana Célia Sousa, Andreia Pereira, Marina Santos, Margarida Temtem, Sónia Freitas, Joel Monteiro, João Adriano Sousa, Ricardo Rodrigues, Graça Guerra, Roberto Palma dos Reis

**Affiliations:** 1 Hospital Central do Funchal, Unidade de Investigação, Serviço de Saúde da Região, SESARAM, EPERAM, Funchal, Portugal.; 2 Universidade Nova de Lisboa, Faculdade de Ciências Médicas, Lisboa, Portugal.

**Keywords:** Traditional risk factors, genetic risk score, events risk discrimination and reclassification, Net Reclassification Index, secondary prevention of coronary artery disease

## Abstract

The inclusion of a genetic risk score (GRS) can modify the risk prediction of coronary artery disease (CAD), providing an advantage over the use of traditional models. The predictive value of the genetic information on the recurrence of major adverse cardiovascular events (MACE) remains controversial. A total of 33 genetic variants previously associated with CAD were genotyped in 1587 CAD patients from the GENEMACOR study. Of these, 18 variants presented an hazard ratio >1, so they were selected to construct a weighted GRS (wGRS). MACE discrimination and reclassiﬁcation were evaluated by C-Statistic, Net Reclassification Index and Integrated Discrimination Improvement methodologies. After the addition of wGRS to traditional predictors, the C-index increased from 0.566 to 0.572 (p=0.0003). Subsequently, adding wGRS to traditional plus clinical risk factors, this model slightly improved from 0.620 to 0.622 but with statistical significance (p=0.004). NRI showed that 17.9% of the cohort was better reclassified when the primary model was associated with wGRS. The Kaplan-Meier estimator showed that, at 15-year follow-up, the group with a higher number of risk alleles had a significantly higher MACE occurrence (p=0.011). In CAD patients, wGRS improved MACE risk prediction, discrimination and reclassification over the conventional factors, providing better cost-effective therapeutic strategies.

## Introduction

Coronary artery disease (CAD) is a complex disease with both environmental and hereditary contributions (Hajar, 2017; Said et al., 2019). CAD is the principal cause of morbidity and mortality worldwide. Therefore, it is crucial, for primary prevention, the prompt identification of individuals with an increased risk of developing this condition (Giampaoli et al., 2005). Similarly to other countries, the evaluation of the absolute risk of CAD in the Madeira Archipelago is based on a combination of risk factors that is the basis of modern CAD prevention. It is estimated that the heritability of CAD is around 40%-60%, and exhaustive research on genetic predisposition could significantly improve the stratification of CAD risk (Watkins and Farrall, 2006; Khera and Kathiseran, 2017). The genetic variants associated with CAD or its risk factors were previously investigated in the GENEMACOR Study (Pereira et al., 2018a). These were derived from candidate gene studies (Tabor et al., 2002; McCarthy et al., 2004), and GWAS published until 2013, some of them with a known physiopathological mechanism, while others remain under investigation (Hirschhorn and Daly, 2005; Wellcome Trust Case Control Consortium, 2007; Hardy and Singleton, 2009; McPherson, 2014). The most relevant genetic variants associated with CAD risk included: *LPA* (rs3798220), *APOE* (rs7412/rs429358), *ADIPOQ* (rs266729), *FTO* (rs8050136), *MC4R* (rs17782313), *ACE* (rs4340), *MTHFR* (rs1801133), *PON1* (rs854560), *TCF21* (rs12190287), *PHACTR1* (rs1332844), *ZC3HC1* (rs11556924) and *CDKN2B-AS1* (rs1333049 and rs4977574) (Pereira *et al.*, 2018b). Incorporating a *multilocus* genetic risk score (GRS) in commonly used clinical tools has previously improved the prediction of CAD risk (Zhao et al., 2017). Models with traditional risk factors (TRF) performed well in multiple cohorts. However, it was suggested that up to 50% of individuals with few risk factors and who had been assigned a low risk of developing the disease were, in fact, CAD patients (Futterman and Lemberg, 1998). Current scientific knowledge supports that genetic information can improve these patients’ risk stratification. This highpoints the potential role of GRS to complement traditional risk prediction strategies as the GRS is independent of age, and acts long before the onset of clinical risk factors (Benson et al., 2018; Dogan et al., 2018). The patients with established cardiovascular disease (CVD) are prone to suffer from subsequent events, including stroke, myocardial infarction, and death. Prior investigation has verified an association between a *multilocus* GRS and recurrent cardiovascular events among CAD patients (Jiang et al., 2020). Whether the genetic information adds value to conventional and clinical risk factors and inﬂuences prognosis remains controversial and inconsistent (Backgr Störk et al., 2006; Patel et al., 2012; Miao et al., 2020).

There is an increasing number of patients living with established CAD due to new pharmacological and interventional therapies (Kandaswamy and Zuo, 2020). 

In this work, we investigated whether the addition of genetic variants to conventional risk factors improves the discrimination and reclassiﬁcation of Major Adverse Cardiovascular Events (MACE) in the context of secondary prevention of CAD in a Portuguese population from GENEMACOR study.

## Subjects and Methods

### Subjects

A total of 1687 consecutive CAD patients were recruited from the Cardiology Department of the Funchal Hospital Centre, which has a regional quality clinical register named “MADEIRA/GESTINTERNMENT”, covering more than 90% of the patients with acute coronary syndrome (ACS) and stable angina (SAP). About 100 patients (5.9%) were lost to follow-up. The remaining 1587 patients with a diagnosis of CAD were included in the study and allocated to two groups: with and without MACE. They were characterized by coronary angiography showing at least one ≥70% stenosis in any of the main coronary arteries (left anterior descending, left circumﬂex, and right coronary artery), previous episodes of acute myocardial infarction, coronary revascularization, unstable angina and chronic angina. The exclusion criteria included valvular heart disease, cardiomyopathy, connective tissue disease, severe liver or renal disease and malignant tumours. Patients with a physical disability were also excluded. This prospective cohort is part of the GENEMACOR study, a case-control study taking part in the Madeira Archipelago, which only includes individuals born and living in the Madeira for at least two generations (therefore considered a homogeneous population) (Brehm *et al*., 2003; Gonçalves *et al*., 2005).

This study followed the Declaration of Helsinki, and it was approved by the Ethics Committee and Institutional board of Funchal Hospital Centre, under protocol number 50/2012. Written informed consent was attained from all subjects at the time of enrolment.

### Cardiovascular outcomes

The cardiovascular outcomes of this work involved a combination of all-cause vascular morbidity and mortality, including recurrent ACS (myocardial infarction and unstable angina), coronary revascularization (percutaneous or surgical coronary intervention), cardiovascular mortality and readmission due to heart failure or ischemic stroke.

Myocardial infarction (MI), according to the universal definition, is a history of typical ischemic chest pain with increased serum levels of creatinine kinase myocardial band (CK-MB) (greater than 1.5 times) and cardiac troponin above the upper limit of normal (Thygesen et al., 2018). Coronary revascularization was any percutaneous coronary intervention or coronary artery bypass graft procedure performed in the absence of myocardial infarction. Unstable angina (UA) was considered when there is an episode of typical discomfort or pain at rest or during more than 10 minutes or two episodes persisting more than five minutes with negative cardiac biomarkers. Alterations in the electrocardiogram, including 0.5 mm ST-segment depression or transient ST-segment elevation or 2 mm T-wave inversion in 2 contiguous leads, may improve this definition specificity of this definition (Braunwald and Morrow, 2013). For cardiovascular mortality, the criteria used is in accordance with the International Classiﬁcation of Diseases 10th Revision (ICD-10) codes I00-I25, I27, I30-I52, and I60-I72. For ischaemic stroke, ICD-10 codes I63 and I64 were adopted. 

In patients with multiple events, only the time of the ﬁrst event was used for further analyses. Patients were followed-up from 13^th^ March 1999 to 5^th^ September 2019. Two cardiologists independently reviewed all prospective and potential outcomes. Confirmation was achieved on Hospital discharge or death-related summary of the events.

### Traditional, laboratory and clinical risk factors for MACE

Covariates of interest include age, gender and the following risk factors:

- Body Mass Index was calculated as weight in kilograms divided by the square of height in meters.
- Smoking status refers to current smokers or subjects having <5 years of cessation (Marston et al., 2014).
- CAD patients were classified as diabetic when taking antidiabetic medication or if their fasting glucose was higher than 126 mg/dL (American Diabetes Association, 2020).
- Dyslipidemia was defined as Low-Density Lipoprotein>140 mg/dL, High-Density Lipoprotein<45 mg/dL for women and <40 mg/dL for men, Triglycerides>150 mg/dL (Catapano et al., 2016).
- CAD family history is considered if one or more close relatives had early CVD: under 55 for men or before 65 for women (Kolber and Scrimshaw, 2014).
- Physical inactivity was considered a risk factor when subjects practised less than 40 minutes per week of moderate physical activity (Physical Activity Guidelines Advisory Committee Report, 2008).
- Arterial hypertension (ATH) was defined as mean blood pressure of over 140 mmHg (systolic), over 90 mmHg (diastolic), or when blood pressure was controlled, if patients were taking antihypertensive drugs (Mancia et al., 2018).
- The consumption of alcohol in grams per week was quantified and considered significant if it was superior to 60 g/week in men and 40 g/week in women. Alcohol abuse was quantified at more than 300 g/week, which corresponds to exceeding two drinks daily (Rehm et al., 1999).
- All laboratory analyses (fasting glucose, total cholesterol, triglycerides, apolipoprotein B (Apo B), lipoprotein (a), homocysteine, C-reactive protein (hsCRP), fibrinogen, leucocytes and hemoglobin) were carried out in the Clinical Pathology Laboratory of the Central Hospital with quality accreditation, based on the Agencia de Calidad Sanitaria de Andalucía (ACSA) Model (international version).
- Heart rate was measured by the number of heart beats per minute (bpm) (Spodick et al., 1992).
- Creatinine (cr) clearance was calculated through the Cockcroft Gault’ formula (Cockcroft and Gault, 1976).
- Left ventricular ejection fraction (LVEF) was measured during cardiac angiography or by two-dimensional echocardiography. It was based on volume estimation as LVEF= [(end-diastolic volume - end-systolic volume) ÷ end-diastolic volume] × 100 (using the apical two- and four-chamber view (Foley et al., 2012).
- Multivessel disease was defined when two or three vessels were affected in contrast to one vessel disease.

### Genetic variants selection and genotyping

Given the large number of genetic variants previously tested in Caucasian populations deriving from multiple studies (candidate genes and GWAS), we only included genes associated with CAD and already investigated in the GENEMACOR study. In total, we considered 33 genetic variants with a Minor Allele Frequency (MAF) > 2% (Kim et al. 2011), which were distributed by five major physiopathological axes (Table S1), according to their most consensual action pathway in coronary atherosclerosis: six single nucleotide polymorphisms (SNPs) in the lipid metabolism axis corresponding to *PSRC1* (rs599839), *PCSK9* (rs2114580), *KIF6* (rs20455), *LPA* (rs3798220), *ZPR1* (rs964184) and *APOE* (rs7412/rs429358); nine SNPs in Diabetes/Obesity and Insulin Resistance axis, namely *ADIPOQ* (rs266729), *IGF2BP2* (rs4402960), *PPARG* (rs1801282), *SLC30A8* (rs1326634), *TCF7L2* (rs7903146), *TAS2R50* (rs1376251), *FTO* (rs8050136), *MC4R* (rs17782313) and *HNF4A* (rs1884613); three SNPs in the Hypertension (Renin-Angiotensin-Aldosterone) axis, namely *AGT* (rs699), *AGT1R* (rs5186) and *ACE* (rs4340); six SNPs were associated with the pro-oxidative state (Oxidation), namely *MTHFR* (rs1801131 and rs1801133), *MTHFD1L* (rs6922269), *PON1* (rs705379, rs662 and rs854560). Finally, nine SNPs whose pathophysiological mechanism is not fully understood and might be involved in cell cycle, genetic transcription, smooth muscle cells differentiation and proliferation or acting as cotransport binders (Cellular): *MIA3* (rs17465637), *GJA4* (rs618675), *TCF21* (rs12190287), *PHACTR1* (rs1332844), *ZC3HC1* (rs11556924), *CDKN2B-AS1* (rs1333049 and rs4977574), *SMAD3* (rs17228212) and *ADAMTS7* (rs3825807) (Assimes and Roberts, 2016) (Table S1).

A TaqMan allelic discrimination assay for genotyping was performed using labelled probes and primers pre-established by the supplier (TaqMan SNP Genotyping Assays, Applied Biosystems). All reactions were done on an Applied Biosystems 7300 Real-Time PCR System and genotypes were determined using the 7300 System SDS Software (Applied Biosystems, Foster City, USA) without any prior knowledge of the individual’s clinical data. Quality check of genotyping techniques was maintained by the inclusion of one non-template control (NTC) in each plate of 96 wells. All SNPs TaqMan assays had blind duplicates accounting for 20% of all samples. Some SNP genotypes were randomly confirmed by conventional direct DNA sequencing, as 10-15% of all samples were re-amplified for sequencing.

### Statistical analysis

*Descriptive and comparative analysis*

Continuous variables were defined as means (±SD) or medians (Q1 - Q3), as appropriate, and categorical variables were determined as frequencies and proportions. We used the t-Student test (or Mann-Whitney) to compare continuous data and χ^2^ tests to compare categorical variables. 

*Construction of wGRS*

For constructing an additive wGRS, 33 SNPs (Table S1) were surveyed for association with MACE in a Cox proportional hazard model. Those SNPs with a Hazard Ratio (HR)>1 were selected for the additive GRS. Subsequently, we counted the number of risk alleles for each of these SNPs, and the genotypes were coded as “0”, “1” and “2” for wild homozygous, heterozygous, and mutated homozygous, respectively. The additive weighted GRS (wGRS) was achieved by summing the product between the HR for each SNP and the number of risk alleles (0, 1, 2).

*WGRS discriminative capacity to predict MACE*

To estimate the wGRS discriminative capacity in MACE prediction, two analysis were performed: firstly, using TRFs as the baseline model, wGRS and clinical risk factors were sequentially added and compared; secondly, using TRFs + clinical risk factors as the baseline, wGRS was included and then compared. Harrell’s C-statistical approach tested the area under the Receiving Operating curve (ROC) of the models with and without wGRS and its statistical significance. C-statistic refers to the probability that predicting the outcome is better than chance comparing Cox regression models. Calibration was verified by Hosmer and Lemeshow goodness-of-fit test.

*MACE reclassification*

Net Reclassification Improvement (NRI) was calculated according to the continuous method. The number of individuals reclassified into higher and lower risk was applied to the two models with and without wGRS. NRI was designated as the percentage of subjects whose risk is changed (upwards or downwards) when adding the new marker (wGRS). Integrated Discrimination Improvement (IDI) can be defined as an increment of the difference between the means of predicted probabilities of two models: with and without the added marker (wGRS) (Steyerberg et al., 2010; Pencina et al., 2012).

*Cumulative hazard rates according to wGRS*

Event-free survival time was defined as the interval between the admission date to the study and the first event of interest. Patients who had not experienced MACE by the time of the last follow-up were censored. Unadjusted survival and its cumulative hazard curves for each of the outcomes were created by Kaplan-Meier estimator. Breslow test was performed to evaluate the differences between the high (higher than the median) and low (lower than the median) risk MACE groups.

The statistics methods used were those of the Statistical Package for the Social Sciences software version 25.0 (IBM, Armonk, NY, USA), MedCalc version 13.3.3.0 and R (version 3.2.0). All *P*-values were two-sided, statistically significant for p<0.05.

## Results

### Study population characteristics: patients with and without MACE

A total of 1587 coronary patients were allocated to two groups: 567 who experienced MACE (35.7%) and 1020 (64.3%) without MACE. MACE included non-fatal myocardial infarction, unstable angina, revascularization (percutaneous coronary intervention PCI and coronary artery bypass grafting CABG), heart failure and ischemic stroke, were documented during the interquartile range (IQR) of 5.7 (min 0.2 - max 20.5) years of follow-up. Of these patients with MACE, 212 (37.4%) resulted in cardiovascular mortality.

The demographic, traditional, laboratory and clinical features of the study population are summarised in Table 1.

**Table 1 -:** Overall characteristics of the study population.

Overall characteristics	Overall (n=1587)	MACE (n=567)	No-MACE (n=1020)	P-value**
Demographic				
Age, years	53.3 ± 7.9	54.0 ± 7.6	52.9 ± 8.1	0.006
Male sex, n (%)	1246 (78.5)	445 (78.5)	801 (78.5)	0.983
BMI, kg/m^2^	28.6 ± 4.3	28.7 ± 4.2	28.6 ± 4.4	0.826
Traditional risk factors				
Smoking status, n (%)	753 (47.4)	241 (42.5)	512 (50.2)	0.003
Diabetes, n (%)	549 (34.6)	236 (41.6)	313 (30.7)	<0.0001
Dyslipidemia, n (%)	1416 (89.2)	520 (91.7)	896 (87.8)	0.017
CAD family history, n (%)	381 (24.0)	144 (25.4)	237 (23.2)	0.334
Physical inactivity, n (%)	1008 (63.5)	407 (71.8)	601 (58.9)	<0.0001
Hypertension, n (%)	1129 (71.1)	431 (76.0)	698 (68.4)	0.001
Alcohol>300gr/week, n (%)	267 (16.8)	121 (21.3)	146 (14.3)	<0.0001
SBP, mmHg	137.6 ± 20.6	137.8 ± 20.7	137.5 ± 20.5	0.763
DBP, mmHg	82.4 ± 11.8	81.6 ± 11.4	82.9 ± 12.0	0.043
Laboratory Risk Factors				
Fasting glucose, mg/dL	106.0 (9.0 - 130.0)	110.0 (98.0 - 138.0)	104.0 (96.0 - 125.0)	<0.0001
Total cholesterol, mg/dL	180.0 (155.0 - 211.0)	184.0 (155.0 - 216.0)	179.0 (154.5 - 207.5)	0.060
LDL, mg/dL	105.1 (83.2 - 127.4)	107.0 (83.6 - 129.4)	104.7 (82.7 - 126.3)	0.212
HDL, mg/dL	42.0 (35.3 - 49.0)	40.0 (34.5 - 48.0)	42.0 (36.0 - 49.0)	0.002
Triglycerides, mg/dL	140.0 (103.0 - 207.0)	146.0 (108.0 - 214.0)	136.0 (101.5 - 202.5)	0.031
Apolipoprotein B, mg/dL	93.4 (77.0 - 111.8)	97.4 (79.8 - 116.6)	93.4 (75.3 - 109.4)	<0.0001
Lipoprotein (a)>30mg/dL, n (%)	620 (39.1)	255 (45.0)	365 (35.8)	<0.0001
Homocysteine>10mg/dL, n (%)	1217 (76.7)	445 (78.5)	772 (75.7)	0.207
CRP>3 mg/L, n (%)	616 (38.8)	293 (51.7)	323 (31.7)	<0.0001
Fibrinogen, mg/dL	388.0 (340.4 - 446.0)	397.0 (342.0 - 462.0)	383.0 (339.5 - 440.0)	0.009
Leucocytes, mg/dL	7.1 (6.1 - 8.4)	7.2 (6.1 - 8.5)	7.1 (6.0 - 8.2)	0.123
Hemoglobin, mg/dL	14.6 (13.8 - 15.3)	14.6 (13.7 - 15.2)	14.6 (13.8 - 15.4)	0.142
Clinical risk factors				
Heart rate, beat/min.	68.6 ± 12.6	70.3 ± 13.6	67.7 ± 11.9	<0.0001
Cr Clearance*<60ml/min, n (%)	123 (7.8)	65 (11.5)	58 (5.7)	<0.0001
Ejection fraction<55, n (%)	450 (28.4)	226 (39.9)	224 (22.0)	<0.0001
Multivessel disease, n (%)	788 (49.7)	361 (63.7)	427 (41.9)	<0.0001
Genetic information				
wGRS	33.4 ± 2.9	33.7 ± 2.8	33.2 ± 2.9	<0.0001

MACE - Major Adverse Cardiovascular Events; BMI - Body mass index; CAD - Coronary artery disease; SBP - Systolic blood pressure; DBP - Diastolic blood pressure; LDL - Low-density lipoprotein; HDL - High-density lipoprotein; CRP - C reactive protein; Cr Clearance - Creatinine Clearance; *Cockroft-Gault<60 ml/min.; wGRS - Weighted genetic risk score; Continuous variables presented as mean ± SD or median (IQR(Q1 - Q3)); **P-values from the Chi-square test for categorical variables and t Student or Mann-Whitney tests for continuous variables, as appropriate; Statistically significant for p<0.05.

Patients who experienced MACE were older than those without, but the percentage of men was similar in both groups. These patients had a more aggressive atherogenic profile when compared to patients who did not suffer MACE. They had a higher prevalence of the some of the most critical risk factors for CAD: diabetes (41.6% vs 30.7%), dyslipidemia (91.7% vs 87.8%), physical inactivity (71.8% vs 58.9%), arterial hypertension (76.0% vs 68.4%) and alcohol intake >300 gr/week (21.3% vs 14.3%) (all p<0.05). Risk factors, such as fasting glucose, triglycerides, apo B, lipoprotein (a) >30mg/dL, CRP>3 mg/L and fibrinogen were presented with higher values in the MACE cohort (p<0.05). Inversely, smoking status and HDL cholesterol were more elevated in the non-MACE group. The variables associated with clinical risk, including heart rate, creatinine clearance <60 ml/min, ejection fraction<55 and multivessel disease, were significantly higher in the cohort of patients with MACE (all p<0.0001) (Table 1).

### Genetic risk for MACE in CAD patients

For the construction of an additive wGRS, 33 SNPs associated with CAD risk from the GENEMACOR study were analysed (Table S1). Although none of these reached statistical significance for MACE risk using a Cox multivariate analysis, 18 SNPs were selected for wGRS since they presented a MACE risk (HR) > 1 (Table 2). These were: *LPA* (rs3798220), *ZPR1* (rs964184), *IGF2BP2* (rs4402960), *TCF7L2* (rs7903146), *TAS2R50* (rs1376251), *FTO* (rs8050136), *MC4R* (rs17782313), *AGT* (rs699), *AGT1R* (rs5186), *MTHFR* (rs1801131), *PON1* (rs705379), *MIA3* (rs17465637), *GJA4* (rs618675), *TCF21* (rs12190287), *PHACTR1* (rs1332844), *ZC3HC1* (rs11556924), *CDKN2B-AS1* (rs1333049 and rs4977574) (Table 2). 

**Table 2 -:** Genetic variants associated with MACE occurrence.

Genetic Variants	Hazard ratio (95% CI)	P-value
*LPA rs3798220*	1.095 (0.909 - 1.320)	0.340
*ZPR1 rs964184*	1.021 (0.875 - 1.191)	0.795
*IGF2BP2 rs4402960*	1.130 (0.992 - 1.287)	0.065
*TCF7L2 rs7903146*	1.018 (0.896 - 1.156)	0.783
*TAS2R50 rs1376251*	1.051 (0.878 - 1.258)	0.587
*FTO rs8050136*	1.074 (0.951 - 1.212)	0.251
*MC4R rs17782313*	1.058 (0.924 - 1.211)	0.413
*AGT rs699*	1.051 (0.934 - 1.184)	0.408
*AGT1R rs5186*	1.085 (0.947 - 1.243)	0.238
*MTHFR rs1801131*	1.045 (0.902 - 1.211)	0.558
*PON1 rs705379*	1.046 (0.910 - 1.203)	0.524
*MIA3 rs17465637*	1.096 (0.959 - 1.253)	0.179
*GJA4 rs618675*	1.018 (0.883 - 1.173)	0.806
*TCF21 rs12190287*	1.130 (0.998 - 1.287)	**0.050**
*PHACTR1 rs1332844*	1.027 (0.912 - 1.158)	0.657
*ZC3HC1 rs11556924*	1.068 (0.939 - 1.215)	0.317
*CDKN2B-AS1 rs1333049*	1.031 (0.784 - 1.357)	0.826
*CDKN2B-AS1 rs4977574*	1.041 (0.789 - 1.374)	0.776

MACE - Major Adverse Cardiovascular Events; CI - Confidence interval;Statistically significant for p<0.05. WGRS calculated by the formula: Σ(HR × Number of risk alleles).

**Figure 1 -:**
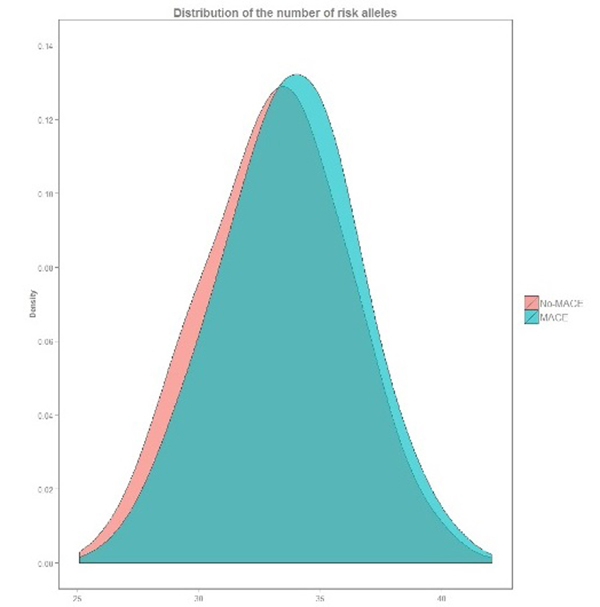
Distribution of the number of risk alleles in CAD patients with and without MACE.

WGRS ranged from 25.1 to 42.1, with a mean of 33.7 ± 2.8 for patients with MACE and 33.2 ± 2.9 for those without MACE (p<0.0001) (Table 1). The curve distribution of wGRS in patients with events (risk allele distribution) is moved to the right of the picture compared to patients’ curve without events (Figure 1).

**Figure 2 ‒:**
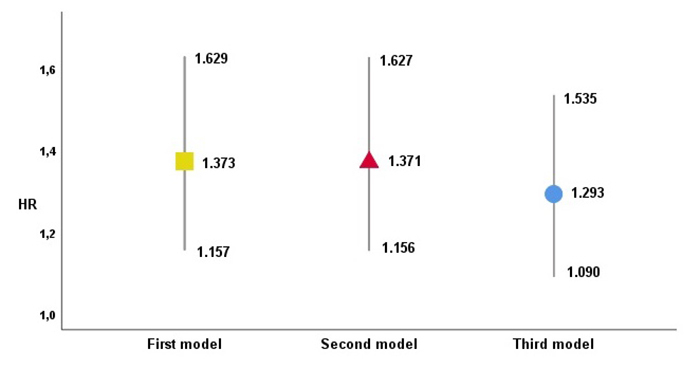
Risk of MACE occurrence with different wGRS adjustments. wGRS Hazard ratios (HR) from Cox regression analysis for prediction of MACE on 3 different models: 1^st^ adjusted for age and gender, 2^nd^ including TRFs and 3^rd^ including TRFs and clinical variables. Statistically significant for each model (p<0.0001; p<0.0001 and p=0.003, respectively)

To evaluate the independent association of the wGRS with the risk of MACE, two groups above and below the median were considered, and three Cox proportional-hazards models were derived. The first was adjusted to age and gender, and the second to the traditional co-variables (gender, age, BMI, smoking, diabetes, dyslipidemia, hypertension and physical inactivity). The third model was adjusted to the same conventional factors plus other clinical co-variables (CRP, ejection fraction, multivessel disease and creatinine clearance). WGRS was significantly associated with MACE risk: the first one with an HR of 1.373, the second with an HR of 1.371 and the third with an HR of 1.293 (Figure 2).

### Comparison of the individual predictive capacity of the different risk factors (traditional, clinical and genetic) to the risk of MACE

We evaluated the individual predictive ability of MACE risk for each traditional, laboratory, clinical and genetic risk factors, using proportional-Hazard Cox multivariate analysis and C-statistic methodology.

After Cox multivariate analysis, all risk factors, except gender, body mass index, dyslipidemia, and smoking, were independently correlated with the occurrence of MACE. The individual HR is exposed in Table 3. It is noted that the wGRS exhibits a more significant correlation to the risk of MACE than dyslipidemia, diabetes, arterial hypertension and decreased renal function. It is only surpassed by CRP> 3mg/L, physical inactivity, low ejection fraction and multivessel disease (Table 3).

**Table 3 -:** Individual MACE risk (HR) of all studied variables.

Overall characteristics	HR (95% CI)	P-value**
Demographic		
Age, years	1.012 (1.002 - 1.023)	0.023
Male sex, n (%)	0.859 (0.703 - 1.050)	0.139
BMI, kg/m^2^	1.009 (0.990 - 1.028)	0.365
Traditional risk factors		
Smoking status, n (%)	0.931 (0.788 - 1.101)	0.405
Diabetes, n (%)	1.300 (1.099 - 1.537)	0.002
Dyslipidemia, n (%)	1.134 (0.841 - 1.529)	0.410
CAD family history, n (%)	0.889 (0.735 - 1.076)	0.226
Physical inactivity, n (%)	1.453 (1.210 - 1.744)	<0.0001
Hypertension, n (%)	1.313 (1.083 - 1.592)	0.006
Alcohol>300gr/week, n (%)	1.374 (1.123 - 1.681)	0.002
SBP, mmHg	0.998 (0.994 - 1.002)	0.348
DBP, mmHg	0.995 (0.988 - 1.002)	0.176
Laboratory Risk Factors		
Fasting glucose, mg/dL	1.002 (1.001 - 1.004)	0.002
Total cholesterol, mg/dL	1.000 (0.998 - 1.002)	0.911
LDL, mg/dL	0.999 (0.997 - 1.001)	0.323
HDL, mg/dL	0.989 (0.982 - 0.997)	0.007
Triglycerides, mg/dL	1.001 (1.000 - 1.001)	0.042
Apolipoprotein B, mg/dL	1.001 (0.998 - 1.003)	0.652
Lipoprotein (a)>30mg/dL, n (%)	1.163 (0.985 - 1.374)	0.074
Homocysteine>10mg/dL, n (%)	1.001 (0.819 - 1.225)	0.989
CRP>3 mg/L, n (%)	1.408 (1.193 - 1.661)	<0.0001
Fibrinogen, mg/dL	1.002 (1.001 - 1.003)	<0.0001
Leucocytes, mg/dL	1.049 (1.010 - 1.089)	0.014
Hemoglobin, mg/dL	0.926 (0.870 - 0.987)	0.017
Clinical risk factors		
Heart rate, beat/min.	1.011 (1.004 - 1.017)	0.001
Cr Clearance*<60ml/min, n (%)	1.349 (1.040 - 1.749)	0.024
Ejection fraction<55, n (%)	1.650 (1.393 - 1.955)	<0.0001
Multivessel disease, n (%)	1.908 (1.608 - 2.265)	<0.0001
Genetic information		
wGRS	1.371 (1.155 - 1.627)	<0.0001

MACE - Major adverse coronary events; HR - Hazard Ratio; CI - Confidence Interval; BMI - Body mass index; CAD - Coronary artery disease; SBP - Systolic blood pressure; DBP - Diastolic blood pressure; LDL - Low density lipoprotein; HDL - High density lipoprotein; CRP - C reactive protein; Cr Clearance - Creatinine Clearance; *Cockroft-Gault<60 ml/min; wGRS -Weighted genetic risk score; **Cox regression analysis entering each variable individually; Statistically significant for p<0.05.

**Figure 3 -:**
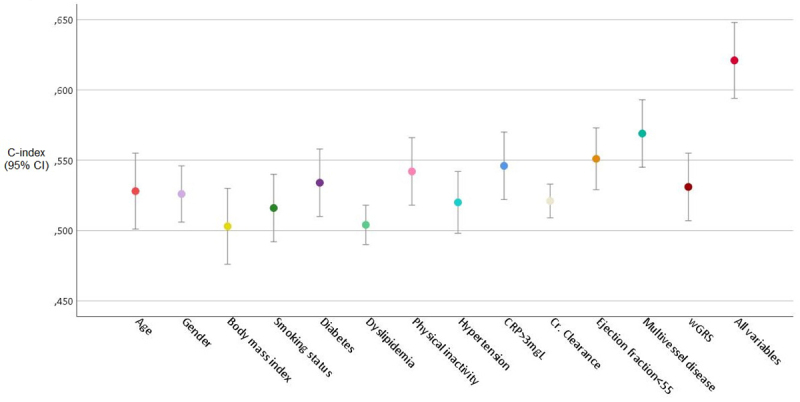
Individual predictive capacity of the investigated variables to MACE risk by C-index methodology.

Using ROC curve for events discrimination (C-statistic), the results are similar to the previous analysis, excepting diabetes, which appears along with the investigated variables yielding a slight improvement for predicting events compared to wGRS (Figure 3).

### Prediction and discrimination of total MACE: models with and without wGRS

For total MACE discrimination, we used a C-Statistic methodology. The model was developed for the composite outcomes of an ACS (MI and UA), revascularization, heart failure, ischemic stroke and death. It was adjusted for all studied traditional and clinical risk factors. When the wGRS was incorporated in the baseline model (with conventional risk factors only), the ROC curve for events discrimination (C-Statistic) increased from 0.566 (95% CI: 0.539-0.593) to 0.572 (95% CI: 0.545-0.599) (p=0.0003). The prognostic C-index was 0.6%. However, when wGRS plus clinical factors were incorporated in the baseline model, it increased from 0.566 to 0.622 (95% CI: 0.595-0.649) (p<0.0001) with a prognostic C-index of 5.6% (Table 4).

**Table 4 -:** MACE discrimination using Harrel’s C-statistic, NRI and IDI methodology.

Models	C-index (95% CI)		P-value
TRFs	0.566 (0.539 - 0.593)		
TRFs + wGRS	0.572 (0.545 - 0.599)		**0.0003^(a)^**
TRFs + clinical + wGRS	0.622 (0.595 - 0.649)		**<0.0001^(b)^**
TRFs + clinical	0.620 (0.593 - 0.647)		
TRFs + clinical + wGRS	0.622 (0.595 - 0.649)		**0.004^(c)^**
		NRI	
	Total	MACE	Non-MACE
TRFs + clinical + wGRS	17.9% (7.9% - 27.9%)	25.9% (18.0% - 33.9%)	-8.0% (-14.2% - (-1.9%))
	**p=0.0005**	**p<0.0001**	**p=0.010**
		IDI	
	0.7% (0.2% - 1.0%)	-	-
	**p=0.002**	-	-

MACE - Major adverse coronary event; NRI - Net reclassification index; IDI - Integrated discrimination index; TRFs - Traditional Risk Factors; wGRS - Weighted genetic risk score; Clinical - Clinical risk factors; CI - Confidence interval. (a) Comparison among TRFs+wGRS vs TRFs models; (b) Comparison between TRFs+clinical+wGRS vs TRFs models; (c) Comparison between TRFs+clinical+wGRS vs TRFs+clinical models. Statistically significant for p<0.05.

Subsequently, adding wGRS to traditional plus clinical risk factors, this model slightly improved from 0.620 to 0.622 but with statistical significance (p=0.004) (Table 4).

### Discrimination and Reclassification of MACE with NRI and IDI methodology

We used the new statistical metrics NRI and IDI to evaluate the progression of discrimination when the model with traditional and clinical information is added to wGRS. The continuous based NRI added to wGRS improved the non-genetic model discrimination, which was confirmed by the IDI. 

Model calibration was tested by goodness-of-fit with the Hosmer and Lemeshow test and was suitable for the model predicting MACE (p=0.461).

Using NRI, the net proportion of events (NRIe) that assigned a higher risk was 25.9% (better reclassified), and the net ratio of non-events (NRIne) was -8.0%. This result reflects an increased percentage of non-events going to a higher category (worst reclassified), resulting in a total NRI of 17.9% of patients being better reclassified when genetic information was included (Table 4). IDI was 0.7% showing a difference in predicted probabilities between patients with and without events.

### Cumulative hazard rates according to wGRS

**Figure 4 ‒:**
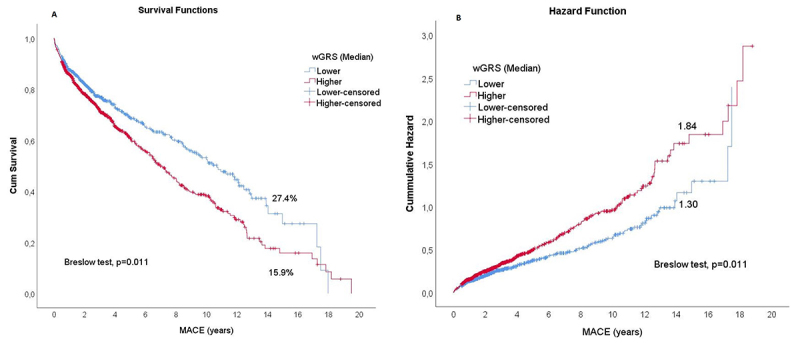
Survival analysis for patients with and without MACE, comparing wGRS above and below the median. (A) Cumulative survival curves: Lower- and higher-censored data are the individuals who, at the time of follow-up, didn’t develop any type of MACE in each wGRS categories (Lower and higher than median). (B) Cumulative hazard curves: Lower- and higher-censored data are the individuals who, at the time of follow-up, didn’t develop any type of MACE in each wGRS categories (Lower and higher than median)

Remarkably, the Kaplan-Meier curve analysis at 15-years follow-up showed the group with a higher number of risk alleles (higher than the median) had a significantly higher occurrence of MACE and worst lifetime (15.9%) when compared to the lower than the median (27.4%) with a fewer number of risk alleles (Figure 4A). The comparison of the two curves was statistically significant with the Breslow test (p=0.011). However, there were no substantial differences between the probability of survival event-free lifetime from the two groups after 18-years of follow-up, and both presented approximately 10% of event-free chance. The cumulative hazard curves showed a higher risk in the group above the median of wGRS (p=0.011) through the years, being similarly at 18-years, approximately (Figure 4B).

## Discussion

In CAD patients, the recurrence of myocardial infarction or other cardiovascular events increases morbidity and mortality. Tools have been developed to predict recurrent or possibly lethal ischemic events, including traditional and clinical risk factors. Unfortunately, risk prediction based on these tools alone is not robust enough, and it was thought that genetic information could improve this ability. Although a prior investigation has shown the predictive value of a *multilocus* genetic risk score to recurrent cardiovascular events in CAD patients, this subject remains controversial and inconsistent (Labos et al., 2015; Smit et al., 2015). As far as we know, the present study is the first in Portugal to investigate the cumulative capacity of genetic variations to predict the risk of MACE occurring over a long period of follow-up in patients with known CAD. Long-term cohorts provide more reliable estimates of survival rates in populations and offer an excellent opportunity to estimate the association between genetic information and specific MACE occurrence in coronary disease.

Given the high burden of recurrent ischemic events and in light of the controversies surrounding this issue, we decided to investigate whether the incorporation of genetic risk factors in a model with traditional and clinical risk factors improved the discriminatory performance of the MACE risk forecast and improved the risk of the patient with CAD installed. We developed an 18-wGRS, based on 33 SNPs previously associated with CAD, being correlated with the risk of adverse cardiovascular events.

This score, tested in three models, was significantly and independently associated with MACE risk.

We have used different methodologies to evaluate our wGRS to discriminate the risk of MACE when incorporated in the traditional risk prediction tools. It was shown that although genetic factors have a significant role in the risk of MACE occurrence, the clinical variables have the highest value in this prediction. C-statistic was not as accurate as the NRI method. A prior simulation study showed the method of testing differences used in C-index is not recommended in situations where the added value of the new predictor is not very significant. Massive associations are required to increase the predictive performance significantly (Delong et al., 1988; Demler et al., 2012). To overcome these drawbacks we applied the indexes (NRI and IDI) proposed by Pencina *et al*. (2012), which more accurately quantify the incremental value of the predictors. The incorporation of genetic information into traditional models, evaluated by NRI and IDI, improved the risk reclassification, which became more discriminatory, accurate and clinically valuable. Specifically, when the genetic information was included in the traditional models (classical plus clinical), 17.9% of our population were better reclassified. These results highlight the importance of considering the addition of a new marker to an established model. NRI methodology, in our opinion, is a more accurate and clinically helpful methodology for quantifying and classifying these patients into different risk groups. Besides, reclassification to a higher risk group allows medical decision-makers to treat and monitor them more aggressively. Genetic information inclusion has more potential to identify patients at high-risk than conventional risk evaluation alone and improve clinical practice (Moorthie et al., 2019). However, the minimal degree of improvement assigned by incorporating the wGRS into established risk tools needed for clinical utility is unknown, and there are still questions about the definitive clinical usefulness of GRS. Additional investigation in high-risk populations incorporating new data from genomics and gene expression studies in developing new genetic risk scores may be helpful. Likewise, new statistical approaches based on data mining and artificial intelligence transform future prediction techniques (Chan et al., 2018).

The cumulative rates of risk events, according to the wGRS, remain controversial. These contradictions can be motivated by different approaches in the evaluation of the results. When the analysis focuses on the initial period, we can get quite different results from those found at the end of an extensive follow-up. In the present study, cumulative risk rate analysis showed that the group with the highest risk alleles was associated with a significantly higher incidence of cardiovascular events and worse event-free lifetime. However, sooner or later, if they live enough, all patients start having events. In the early stages of life, genetic susceptibility to CAD (heritability) has a significant impact, but this propensity “loses traction” over time, as it competes with lifestyle and environmental factors, medications, and other comorbidities. Some initially lower-risk individuals, over an extended follow-up, get older, and degenerative CAD mechanisms go forward exhausting all therapeutic options for chronic CVD (statins, antihypertensives, and revascularization) starting to behave in a similar way to higher-risk patients. So, at the end of a long follow-up the two survival curves overlap.

Our work´s GRS emphasizes the concept that genetic information can be more useful in early life, stratifying individuals with different atherosclerotic profiles. Inouye et al. (2018) proposed that patients with increased genetic risk could obtain the maximum benefit from therapies with early intervention in hyperlipidemia and hypertension, generating a subpopulation that benefits from cost-effective primary prevention.

Except for diabetics, there is insufficient research about the association between combined genetic variants and the incidence of major cardiovascular events in patients with known CAD (Sousa et al., 2011; Zhao et al., 2017). The association between these 18 *loci* and MACE has been established. It is unknown which of these *loci* has a more significant contribution to coronary events in existing CAD. Variants leading to acute coronary events may diverge from those influencing recurrent MACE since they predispose to plaque instability rather than its formation.

Of the studied 18 genetic variants presenting an HR>1, the gene that came closest to statistical significance (p=0.05) was TCF21 rs12190287 and should be further tested in other works as a potential predictor for adverse events (Nurnberg et al., 2015).

Genetic scores could allow for precision approaches in medicine, categorizing patient subgroups at increased risk of recurrent events or those with specific pathophysiology in which a particular targeted therapy or preventive strategy would be more beneficial. Current studies strongly argue that CAD risk is proportionate to the duration and strength of exposure to hyperlipidemia. Navar-Bogan *et al*. (2015), based on the Framingham Offspring Cohort, show a two-times increase in CAD risk and cardiac events for each decade of hyperlipidemia exposure (Navar-Bogan **et al.** , 2015; Roberts et al., 2020). Over the subsequent years, the advances in human genomics and proteomics may well allow for identifying protein biomarkers that predict cardiovascular outcomes, including biomarkers involved in regulating homeostasis and inflammatory pathways. Using a targeted proteomics platform, we can extend previous and established novel biomarker associations with the incidence of CVD events, validating past genetic associations (Ho et al., 2018). When genetic sequencing becomes more affordable, as expected, new data can guide efforts in the development of safer, more effective drugs and in helping providers to prevent and treat CVD.

## Strengths and limitations

This study´s main strength is the homogeneity of the population, all born and living in a genetically isolated area: Madeira Archipelago. It is a Southern European Caucasian population with no genetic admixtures. Most studies with GRS included thousands of individuals and samples from different cohorts with heterogeneous people, which can be criticized because this heterogeneity can impact the genetic study’s results.

Another strength is that this study was explicitly designed to detention a representative population of coronary patients, who had been carefully characterized at baseline, and the predictors have been selected using both clinical and statistical awareness.

Thirdly, our study had an extended prospective follow-up, allowing us to study the inﬂuence of MACE risk variants on long-term outcomes. It is recognized how difficult it can be to collect and record detailed information about such a large number of patients during an extended follow-up. 

Our results must also be taken in the light of possible limitations. Our GRS comprised a group of SNPs whose pathophysiological mechanism may be associated with a clinically relevant increase in the risk of MACE. However, if it included many SNPs associated with different pathophysiological axes, it could be more informative. Future research in genomics, proteomics and gene expression may reveal new markers associated with inflammation, endothelium, thrombosis and new environmental triggers, which help to unravel the complexity of CAD and establish the bases of precision medicine in this field (Eagle *et al*., 2010; Brunicardi *et al*., 2011; Leopold and Loscalzo, 2018).

Another major limitation of our study is the sample size. Our findings need to be validated in a larger sample of patients with chronic CAD. 

In conclusion: Our wGRS improved CVD risk discrimination and reclassification over and above the conventional risk factors. Besides, we demonstrate that wGRS is more useful in early life, in addition to traditional and clinical risk factors. Further GRS screening could help specifically patients at intermediate risk for cardiovascular events to prevent future episodes by intensive statin and antihypertensive treatments. 

## Supplementary Material

Table S1 ‒ Genetic variants associated with CAD risk in the GENEMACOR population (n=3120)

## References

[B1] American Diabetes Association (2020). Glycemic targets: Standards of medical care in
diabetes. Diabetes Care.

[B2] Assimes TL, Roberts R (2016). Genetics: Implications for prevention and management of coronary
artery disease. J Am Coll Cardiol.

[B3] Backgr Störk S, Feelders RA, van den Beld AW, Steyerberg EW, Savelkoul HF, Lamberts SW, Grobbee DE, Bots ML (2006). Prediction of mortality risk in the elderly. Am J Med.

[B4] Benson MD, Yang Q, Ngo D, Zhu Y, Shen D, Farrell LA, Sinha S, Keyes MJ, Vasan RS, Larson MG (2018). The Genetic architecture of the cardiovascular risk
proteome. Circulation.

[B5] Braunwald E, Morrow DA (2013). Unstable Angina: Is it time for a requiem?. Circulation.

[B6] Brehm A, Pereira L, Kivisild T, Amorim A (2003). Mitochondrial 00161 portraits of the Madeira and Açores
archipelagos witness different genetic pools of its settlers. Hum Genet.

[B7] Brunicardi FC, Gibbs RA, Wheeler DA, Nemunaitis J, Fisher W, Goss J, Chen C (2011). Overview of the development of personalized genomic medicine and
surgery. World J Surg.

[B8] Catapano AL, Graham I, De Backer G, Wiklund O, Chapman MJ, Drexel H, Hoes AW, Jennings CS, Landmesser U, Pedersen TR (2016). 2016 ESC/EAS guidelines for the management of
dyslipidaemias. Eur Heart J.

[B9] Chan YK, Chen Y-F, Pham T, Chang W, Hsieh M-Y (2018). Artificial intelligence in medical applications. J Healthc Eng.

[B10] Cockcroft DW, Gault MH (1976). Prediction of creatinine clearance from serum
creatinine. Nephron.

[B11] DeLong ER, DeLong DM, Clarke-Pearson DL (1988). Comparing the areas under two or more correlated receiver
operating characteristic curves: a nonparametric approach. Biometrics.

[B12] Demler OV, Pencina MJ, D’Agostino RB Sr (2012). Misuse of DeLong test to compare AUCs for nested
models. Stat Med.

[B13] Dogan MV, Grumbach IM, Michaelson JJ, Philibert RA (2018). Integrated genetic and epigenetic prediction of coronary heart
disease in the Framingham Heart Study. PLoS One.

[B14] Eagle KA, Ginsburg GS, Musunuru K, Aird WC, Balaban RS, Bennett SK, Blumenthal RS, Coughlin SR, Davidson KW, Frohlich ED (2010). Identifying patients at high risk of a cardiovascular event in
the near future: Current status and future directions - Report of a National
Heart, Lung, and Blood Institute. Circulation.

[B15] Foley TA, Mankad SV, Anavekar NS, Bonnichsen CR, Morris MF, Miller TD, Araoz PA (2012). Measuring left ventricular ejection fraction - techniques and
potential pitfalls. Eur Cardiol.

[B16] Futterman LG, Lemberg L (1998). Fifty per cent of patients with coronary artery disease do not
have any of the conventional risk factors. Am J Crit Care.

[B17] Giampaoli S, Palmieri L, Mattiello A, Panico S (2005). Definition of high-risk individuals to optimise strategies for
primary prevention of cardiovascular diseases. Nutr Metab Cardiovasc Dis.

[B18] Gonçalves R, Freitas A, Branco M, Rosa A, Fernandes AT, Zhivotovsky LA, Underhill PA, Kivisild T, Brehm A (2005). Y-chromosome lineages from Portugal, Madeira and Açores record
elements of Sephardim and Berber ancestry. Ann Hum Genet.

[B19] Hajar R (2017). Risk factors for coronary artery disease: Historical
perspectives. Heart Views.

[B21] Hardy J, Singleton A (2009). Genome-wide association studies and human disease. N Engl J Med.

[B22] Hirschhorn JN, Daly MJ (2005). Genome-wide association studies for common diseases and complex
traits. Nat Rev Genet.

[B23] Ho JE, Lyass A, Courchesne P, Chen G, Liu C, Yin X, Hwang S-J, Massaro JM, Larson MG, Levy D (2018). Protein biomarkers of cardiovascular disease and mortality in the
community. J Am Heart Assoc.

[B24] Inouye M, Abraham G, Nelson CP, Wood AM, Sweeting MJ, Dudbridge F, Lai FY, Kaptoge S, Brozynska M, Wang T (2018). Genomic risk prediction of coronary artery disease in 480,000
adults implications for primary prevention. J Am Coll Cardiol.

[B25] Jiang J, Zheng Q, Han Y, Qiao S, Chen J, Yuan Z, Yu B, Ge L, Jia J, Gong Y (2020). Genetic predisposition to coronary artery disease is predictive
of recurrent events: A Chinese prospective cohort study. Hum Mol Genet.

[B26] Kandaswamy E, Zuo L (2018). Recent advances in treatment of coronary artery disease: Role of
science and technology. Int J Mol Sci.

[B27] Khera AV, Kathiresan S (2017). Genetics of coronary artery disease: discovery, biology and
clinical translation. Nat Rev Genet.

[B28] Kolber MR, Scrimshaw C (2014). Family history of cardiovascular disease. Can Fam Physician.

[B29] Kim SY, Lohmueller KE, Albrechtsen A, Li Y, Korneliussen T, Tian G, Grarup N, Jiang T, Andersen G, Witte D (2011). Estimation of allele frequency and association mapping using
next-generation sequencing data. BMC Bioinformatics.

[B30] Labos C, Martinez SC, Wang RH, Lenzini PA, Pilote L, Bogaty P, Brophy JM, Engert JC, Cresci S, Thanassoulis G (2015). Utility of a genetic risk score to predict recurrent
cardiovascular events 1 year after an acute coronary syndrome: A pooled
analysis of the RISCA, PRAXY, and TRIUMPH Cohorts. Atherosclerosis.

[B31] Leopold JA, Loscalzo J (2018). The emerging role of precision medicine in cardiovascular
disease. Circ Res.

[B32] Mancia G, Rosei EA, Azizi M, Burnier M, Clement DL, Coca A, Simone G, Dominiczak A, Kahan T, Mahfoud F (2018). ESC/ESH Guidelines for the management of arterial hypertension:
The task force for the management of arterial hypertension of the European
Society of Cardiology (ESC) and the European Society of Hypertension
(ESH). Eur Heart J.

[B33] Marston L, Carpenter JR, Walters KR, Morris RW, Nazareth I, White IR, Petersen I (2014). Smoker, ex-smoker or non-smoker? The validity of routinely
recorded smoking status in UK primary care: a cross-sectional
study. BMJ Open.

[B34] McCarthy J, Parker A, Salem R, Moliterno DJ, Wang Q, Plow EF, Rao S, Shen G, Rogers WJ, Newby LK (2004). Large scale association analysis for identification of genes
underlying premature coronary heart disease: cumulative perspective from
analysis of 111 candidate genes candidates. J Med Genet.

[B35] McPherson R (2014). Genome-Wide association studies of cardiovascular disease in
European and Non-European Populations. Curr Genet Med Rep.

[B36] Miao B, Hernandez AV, Alberts MJ, Mangiafico N, Roman YM, Coleman CI (2020). Incidence predictors of Major Adverse Cardiovascular Events in
patients with established atherosclerotic disease or multiple risk
factors. J Am Heart Assoc.

[B37] Moorthie S, Villiers CB, Brigden T, Gaynor L, Hall A, Johnson E, Sanderson S, Kroese M (2019). Polygenic scores, risk and cardiovascular disease.

[B38] Navar-Boggan AM, Peterson ED, D’Agostino RB, Neely B, Sniderman AD, Pencina MJ (2015). Hyperlipidemia in early adulthood increases long-term risk of
coronary heart disease. Circulation.

[B39] Nurnberg ST, Cheng K, Raiesdana A, Kundu R, Miller CL, Kim JB, Arora K, Carcamo-Oribe I, Xiong Y, Tellakula N (2015). Coronary artery disease associated transcription factor TCF21
regulates smooth muscle precursor cells that contribute to the fibrous
cap. PLoS Genet.

[B40] Patel RS, Sun YV, Hartiala J, Veledar E, Su S, Sher S, Liu YX, Rahman A, Patel R, Rab ST (2012). Association of a genetic risk score with prevalent and incident
myocardial infarction in subjects undergoing coronary
angiography. Circ Cardiovasc Genet.

[B41] Pencina MJ, D’Agostino RB, Pencina KM, Janssens AC, Greenland P (2012). Interpreting the incremental value of markers added to risk
prediction models. Am J Epidemiol.

[B42] Pereira A, Mendonça MI, Borges S, Freitas S, Henriques E, Rodrigues M, Freitas AI, Sousa AC, Brehm A, Palma dos Reis R (2018). Genetic risk analysis of coronary artery disease in a population
based study in Portugal, using a genetic risk score of 31
variants. Arq Bras Cardiol.

[B43] Pereira A, Mendonca MI, Borges S, Sousa AC, Freitas S, Henriques E, Rodrigues M, Freitas AI, Guerra G, Freitas C (2018). Additional value of a combined genetic risk score to standard
cardiovascular stratification. Genet Mol Biol.

[B44] Physical Activity Guidelines Advisory Committee Report, 2008. To the
Secretary of Health and Human Services (2009). Part A: executive summary. Nutr Rev.

[B45] Rehm J, Greenfield TK, Walsh G, Robson L, Single E (1999). Assessment methods for alcohol consumption, prevalence of
high-risk drinking and harm: A sensitivity analysis. Int J Epidemiol.

[B46] Roberts R, Campillo A, Schmitt M (2020). Prediction and management of CAD risk based on genetic
stratification. Trends Cardiovasc Med.

[B47] Said MA, van de Vegte YJ, Zafar MM, van der Ende MY, Raja GK, Verweij N, van der Harst P (2019). Contributions of interactions between lifestyle and genetics on
coronary artery disease risk. Curr Cardiol Rep.

[B48] Smit JA, Ware EB, Middha P, Beacher L, Kardia SL (2015). Current applications of genetic risk scores to cardiovascular
outcomes and subclinical phenotypes. Curr Epidemiol Rep.

[B49] Sousa AG, Lopes NH, Whady AH, Krieger JE, Pereira AC (2011). Genetic variants of diabetes risk and incident cardiovascular
events in chronic coronary artery disease. PLoS One.

[B50] Spodick DH, Raju P, Bishop RL, Rifkin RD (1992). Operational definition of normal sinus heart rate. Am J Cardiol.

[B51] Steyerberg EW, Vickers AJ, Cook NR, Gerds T, Gonen M, Obuchowski N, Pencina MJ, Kattan MW (2010). Assessing the performance of prediction models: a framework for
some traditional and novel measures. Epidemiology.

[B52] Tabor HK, Risch NJ, Myers RM (2002). Candidate gene approaches for studying complex genetic traits:
practical considerations. Nat Rev Genet.

[B53] Thygesen K, Alpert JS, Jaffe AS, Chaitman BR, Bax JJ, Morrow DA, White HD, Executive Group on behalf of the Joint European Society of
Cardiology (ESC), American College of Cardiology (ACC), American Heart Association (AHA), World Heart Federation (WHF), Task Force for the Universal Definition of Myocardial
Infarction (2018). Fourth Universal Definition of Myocardial
Infarction. J Am Coll Cardiol.

[B54] Watkins H, Farrall M (2006). Genetic susceptibility to coronary artery disease: from promise
to progress. Nat Rev Genet.

[B55] Wellcome Trust Case Control Consortium (2007). Genome-wide association study of 14,000 cases of seven common
diseases and 3,000 shared controls. Nature.

[B56] Zhao C, Zhu P, Shen Q, Jin L (2017). Prospective association of a genetic risk score with major
adverse cardiovascular events in patients with coronary artery
disease. Medicine.

