# Supplementary material for: Genetic information improves the prediction of major adverse cardiovascular events in the GENEMACOR population
Source: Genet Mol Biol. 2021 Jun 11;44(2):e20200448. doi: 10.1590/1678-4685-GMB-2020-0448 (PMC8201463; doi:10.1590/1678-4685-GMB-2020-0448)
Supplement: Table S1 ‒ [file 1415-4757-GMB-44-2-e20200448-s1.pdf]

Genetics and Molecular Biology
DOI: https://doi.org/10.1590/1678-4685-GMB-2020-0448

Supplementary Material to "Genetic information improves the prediction of major adverse cardiovascular events in the GENEMACOR population"

Table S1 - Genetic variants associated with CAD risk in the GENEMACOR population (n=3120).

SNP ID	NEAREST GENE	ALLELES	CHR	GENOTYPIC OR (95% CI)	P VALUE	ALLELIC OR (95% CI)	P VALUE	MAF (%)	PUTATIVE FUNCTION*
rs599839	*PSRC1*	G>A	1	1.167 (0.825-1.652)[#]	0.382	1.043 (0.924-1.178)	0.493	21.3	Lipid metabolism
rs2114580	*PCSK9*	A>G	1	1.068 (0.927-1.230)[*]	0.363	1.029 (0.919-1.152)	0.619	26.4	Lipid metabolism
rs20455	*KIF6*	T>C	6	1.153 (0.922-1.441)[*]	0.211	1.059 (0.953-1.178)	0.287	33.0	Lipid metabolism
rs3798220	*LPA*	T>C	6	1.461 (1.200-1.780)[+]	*<0.0001*	2.105 (1.424-3.113)	*0.0001*	2.0	Lipid metabolism
rs964184	*ZPR1*	C>G	11	1.109 (0.971-1.266)[+]	0.126	1.107 (0.971-1.262)	0.129	17.6	Lipid metabolism
rs7412/rs429358[1]	*APOE*[1]	ε4	19	1.264 (1.072-1.491)[#]	*0.005*	1.237 (1.068-1.434)	*0.005*	13.4	Lipid metabolism
rs266729	*ADIPOQ*	C>G	3	1.137 (1.011-1.280)[•]	*0.033*	1.137 (1.011-1.280)	*0.033*	23.4	Diabetes/Obesity
rs4402960	*IGF2BP2*	G>T	3	1.111 (0.872-1.414)[*]	0.394	0.999 (0.897-1.113)	0.987	30.6	Diabetes/Obesity
rs1801282	*PPARG*	C>G	3	1.144 (0.960-1.364)[•]	0.133	1.144 (0.960-1.364)	0.133	8.7	Diabetes/Obesity
rs1326634	*SLC30A8*	T>C	9	1.159 (0.883-1.520)[#]	0.287	1.060 (0.947-1.188)	0.311	26.0	Diabetes/Obesity
rs7903146	*TCF7L2*	C>T	10	1.011 (0.815-1.254)[#]	0.919	0.999 (0.900-1.109)	0.987	35.1	Diabetes/Obesity
rs1376251	*TAS2R50*	G>A	12	1.224 (0.644-2.326)[*]	0.536	1.032 (0.885-1.203)	0.690	12.0	Diabetes/Obesity
rs8050136	*FTO*	C>A	16	1.186 (1.025-1.373)[#]	*0.022*	1.125 (1.016-1.246)	*0.023*	39.7	Diabetes/Obesity
rs17782313	*MC4R*	T>C	18	1.387 (1.000-1.925)[*]	*0.049*	1.071 (0.949-1.209)	0.268	21.7	Diabetes/Obesity
rs1884613	*HNF4A*	C>G	20	0.880 (0.754-1.027)[*]	0.104	0.921 (0.804-1.054)	0.232	16.2	Diabetes/Obesity
rs699	*AGT*	T>C	1	0.954 (0.822-1.109)[#]	0.542	0.991 (0.896-1.096)	0.860	42.7	Hypertension
rs5186	*AGT1R*	A>C	3	1.080 (0.961-1.213)[+]	0.198	1.078 (0.961-1.210)	0.202	24.9	Hypertension
rs4340	*ACE*	I>D	17	1.170 (1.012-1.354)[*]	*0.034*	1.087 (0.981-1.204)	0.111	38.2	Hypertension
rs1801131	*MTHFR*	A>C	1	1.085 (0.942-1.249)[*]	0.259	1.064 (0.952-1.188)	0.273	28.3	Oxidation
rs1801133	*MTHFR*	C>T	1	1.208 (1.049-1.392)[#]	*0.009*	1.135 (1.021-1.261)	*0.019*	33.4	Oxidation
rs6922269	*MTHFD1L*	G>A	6	0.969 (0.842-1.116)[#]	0.666	0.988 (0.883-1.104)	0.828	27.3	Oxidation
rs705379	*PON1*	C>T	7	1.088 (0.919-1.288)[#]	0.325	1.044 (0.945-1.154)	0.393	46.8	Oxidation
rs662	*PON1*	A>G	7	1.154 (0.907-1.466)[#]	0.243	1.061 (0.952-1.182)	0.287	30.0	Oxidation
rs854560	*PON1*	T>A	7	1.147 (1.036-1.270)[•]	*0.008*	1.147 (1.036-1.270)	*0.008*	40.6	Oxidation
rs17465637	*MIA3*	A>C	1	1.083 (0.970-1.208)[•]	0.157	1.083 (0.970-1.208)	0.157	28.8	Cellular
rs618675	*GJA4*	T>C	1	1.155 (0.807-1.654)[*]	0.431	1.041 (0.918-1.180)	0.530	19.6	Cellular
rs12190287	*TCF21*	G>C	6	1.202 (1.080-1.338)[+]	*0.001*	1.199 (1.079-1.333)	*0.001*	33.0	Cellular

SNP ID	NEAREST GENE	ALLELES	CHR	GENOTYPIC OR (95% CI)	P VALUE	ALLELIC OR (95% CI)	P VALUE	MAF (%)	PUTATIVE FUNCTION*
rs1332844	*PHACTR1*	C>T	6	1.183 (1.016-1.379)[*]	*0.031*	1.115 (1.009-1.232)	*0.033*	44.5	Cellular
rs11556924	*ZC3HC1*	T>C	7	1.142 (1.028-1.270)[+]	*0.013*	1.141 (1.027-1.267)	*0.014*	34.2	Cellular
rs1333049	*CDKN2B-AS1*	G>C	9	1.134 (1.026-1.253)[•]	*0.014*	1.134 (1.026-1.253)	*0.014*	45.6	Cellular
rs4977574	*CDKN2B-AS1*	A>G	9	1.152 (1.041-1.274)[•]	*0.006*	1.152 (1.041-1.274)	*0.006*	41.9	Cellular
rs17228212	*SMAD3*	T>C	15	1.121 (0.840-1.496)[*]	0.436	1.009 (0.900-1.132)	0.873	25.5	Cellular
rs3825807	*ADAMTS7*	A>G	15	0.937 (0.847-1.037)[•]	0.207	0.937 (0.847-1.037)	0.207	41.3	Cellular

SNP – Single Nucleotide Polymorphism; Chr – Chromosome; OR – Odds Ratio; CI – Confidence Interval; MAF – Minor Allele Frequency; [+]Additive model;*Recessive model; [#]Dominant model; [•]Allelic model; [1]Resulting from a Haplotype; Table shows susceptibility loci for CAD, genotypic and allelic ORs and p values for the lead SNP within each locus from GWAS and candidate gene studies. Genotypic ORs are given for additive, recessive, allelic or dominant model. Potential mechanism of action is based on what is already known about the function of the nearby genes. It includes Lipid metabolism, Diabetes/Obesity, Hypertension, Oxidation (genes involved in pro-oxidative status) and Cellular (genes associated to cell cycle, cellular migration and inflammation). *Themistocles L. Assimes, Robert Roberts. JACC, Vol. 68; (25); 2016.
